# Understanding the Mechanism of Action of the Anti-Dandruff Agent Zinc Pyrithione against *Malassezia restricta*

**DOI:** 10.1038/s41598-018-30588-2

**Published:** 2018-08-14

**Authors:** Minji Park, Yong-Joon Cho, Yang Won Lee, Won Hee Jung

**Affiliations:** 1 Department of Systems Biotechnology, Chung-Ang University, Anseong, 17546 Korea; 2 Korea Polar Research Institute, Incheon, 21990 Korea; 3 Department of Dermatology, School of Medicine, Konkuk University, Seoul, 05029 Korea; 4 Research Institute of Medicine, Konkuk University, Seoul, 05029 Korea

## Abstract

Dandruff is known to be associated with *Malassezia restricta*. Zinc pyrithione (ZPT) has been used as an ingredient in anti-dandruff treatments. The mechanism of ZPT has been investigated in several studies; however, a non-pathogenic model yeast, such as *Saccharomyces cerevisiae* was most often used. The aim of the present study was to understand how ZPT inhibits the growth of *M. restricta*. We analyzed the cellular metal content and transcriptome profile of ZPT-treated *M. restricta* cells and found that ZPT treatment dramatically increased cellular zinc levels, along with a small increase in cellular copper levels. Moreover, our transcriptome analysis showed that ZPT inhibits Fe-S cluster synthesis in *M. restricta*. We also observed that ZPT treatment significantly reduced the expression of lipases, whose activities contribute to the survival and virulence of *M. restricta* on human skin. Therefore, the results of our study suggest that at least three inhibitory mechanisms are associated with the action of ZPT against *M. restricta*: (i) an increase in cellular zinc levels, (ii) inhibition of mitochondrial function, and (iii) a decrease in lipase expression.

## Introduction

Dandruff is a common scalp condition associated with abnormal scalp flaking. Half of the adult human population is affected by the condition at some time in their lives^1^. In general, dandruff is ascribed to three etiological factors, *Malassezia* yeast, sebaceous secretions, and individual susceptibility^2^. Among these, *Malassezia* yeast has long been considered the main cause of dandruff; this idea is supported by improvement in dandruff, accompanied by a reduction in the number of the yeast cells on the scalp, following treatment with a shampoo containing an antifungal agent^3–5^. A total of 17 different *Malassezia* species have been identified, and, among them, *M. restricta* is the dominant species on human skin^6–10^. Moreover, recent large-scale sequencing analyses have indicated increased presence of *M. restricta* on scalps with dandruff compared to that on healthy scalps, suggesting an association between the fungus and dandruff  ^8,11,12^. The studies have suggested that lipases secreted from *Malassezia* contribute to the development of dandruff and led to the hypothesis that these enzymes hydrolyze sebum triglycerides and help the yeast cells take up saturated fatty acids to generate energy. The accumulation of excess unsaturated fatty acids, such as oleic acid, on the skin causes skin irritation in patients suffering from dandruff^2,13^.

Zinc pyrithione is a derivative of pyrithione (1-hydroxy-2-pyridinethione), which is synthesized from the antimicrobial metabolite ‘aspergillic acid’ of *Aspergillus flavus*^14–17^. A number of shampoos and rinse-off products containing 0.3–2% ZPT have been used extensively over the counter to treat dandruff^18^. Although the use of ZPT as an antidandruff agent is widespread, its mechanism of action against *Malassezia*, and *M. restricta* in particular, is still unclear; only physiological observations of *Malassezia* growth inhibition by ZPT have been reported^19,20^. The mechanism of action of ZPT has been characterized using different model fungal organisms, rather than *M. restricta*. Ermolayeva and Sanders^21^ used *Neurospora crassa* and suggested that ZPT leads to membrane depolarization either directly or indirectly, thereby, inhibiting proton pump-mediated membrane transport. They suggested that this inhibition might be caused solely by pyrithione, because zinc salt is believed to dissociate after ZPT is transported into the cytosol, and that pyrithione alone acted in this manner^21,22^. However, membrane depolarization occurs at a significantly higher concentration of pyrithione, which is beyond the inhibitory concentration for the fungi^23^; therefore, it is difficult to assert that the mechanism of action of ZPT on fungi involves membrane depolarization.

Yasokawa *et al*.^24^ analyzed the transcriptome of *Saccharomyces cerevisiae* cells treated with ZPT and found that the compound upregulates the expression of genes required for iron transport. These results suggested that ZPT induces iron starvation in the yeast cells. Furthermore, they observed that the addition of iron restored the growth of *S. cerevisiae* cells in medium containing ZPT, supporting the idea that the compound induces iron deficiency in the yeast. In contrast, another study by Reeder *et al*.^25^ showed that iron levels in ZPT-treated *S. cerevisiae* cells were not changed, although they observed an upregulation of expression of Fet3, a ferroxidase in the high-affinity reductive iron transport system, which suggested that iron starvation might not be a direct cause of ZPT toxicity. Instead, Reeder *et al*.^25^ found that cellular copper levels were increased on ZPT treatment and observed a downregulation of *CTR1*, a high-affinity copper transporter in the plasma membrane. To confirm that increased cellular copper levels are one of the main causes of growth inhibition by ZPT, a *S. cerevisiae* haploid gene deletion library was screened against ZPT. Deletion mutants that were growth inhibited upon ZPT treatment included a mutant lacking *ACE1*, which encodes a transcription factor responsible for detoxification of high cellular copper levels, a finding of which supports the idea that ZPT treatment causes copper toxicity in yeast cells. Moreover, a number of deletion mutants that lack genes involved in iron-sulfur cluster (Fe-S) assembly in mitochondria showed significant growth defects following ZPT treatment. These results suggested that, in addition to copper toxicity, copper-mediated inactivation of Fe-S cluster assembly in mitochondria also contributes to growth inhibition by ZPT, at least in *S. cerevisiae*.

Although previous studies have suggested possible mechanisms of action of ZPT against model fungal organisms, they have not clearly explained how ZPT inhibits the growth of *Malassezia*, which is phylogenetically distant. Therefore, in this study, we attempted to understand the mechanism of action of ZPT against *M. restricta*, using biochemical and transcriptome analyses and found that ZPT mainly triggered zinc toxicity and mitochondrial dysfunction. Furthermore, our data suggested that ZPT decreases expression of lipases, which may play an important role in survival of the *M. restricta* on the surfaces of scalps of patients with dandruff^13,26,27^. To our knowledge, this is the first comprehensive study to directly investigate the mechanism of action of ZPT against *M. restricta*.

## Results

### ZPT treatment led to an increase in cellular zinc levels in *M. restricta*

ZPT is a well-known zinc ionophore that has antifungal activity, and several studies using model fungi have indicated that alteration of cellular metal content is one of the main antifungal mechanisms of action of the compound. Examples include an increase in cellular copper levels in ZPT-treated *S. cerevisiae*, as mentioned previously. We, therefore, investigated how ZPT treatment altered cellular levels of essential metals, such as copper, zinc, manganese, and iron. The metal content of ZPT-treated *S. cerevisiae* cells was measured in parallel with inductively coupled plasma-atomic emission spectroscopy (ICP-AES) to replicate data previously reported by Reeder *et al*.^25^. The results were similar to what was previously observed (see Supplementary Table S1), confirming that ZPT increases cellular copper levels in *S. cerevisiae*. Furthermore, the results indicated that our method to determine cellular metal levels was reliable.

**Figure 1:**
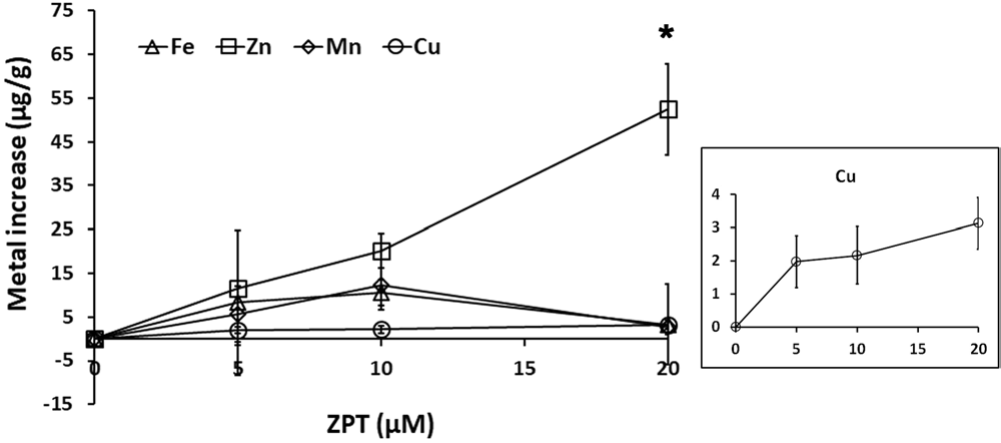
Metal content of ZPT-treated *M. restricta* cells. The iron, copper, zinc, and manganese levels in *M. restricta* KCTC 27527 cells grown in media containing different concentrations of ZPT were determined by ICP analysis. Values represent the average from three independent experiments with standard deviations (*p < 0.005). Changes in copper levels are shown separately.

Subsequently, cellular levels of essential metals in *M. restricta* cells grown in the presence of ZPT were determined by the same method used to investigate whether ZPT altered cellular metal levels in the fungus, and the results showed that, among the essential metals, cellular zinc levels were significantly and dose-dependently increased in cells grown in the presence of ZPT. However, unlike *S. cerevisiae*, only a small increase in copper levels was observed in cells grown in the presence of ZPT compared to that in cells grown in the absence of the compound, and no change in iron or manganese levels was detected (Fig. 1). These data suggest that the mechanism by which ZPT affects the metal content in *M. restricta* is different from that in *S. cerevisiae*, and that ZPT mainly increased zinc levels along with small increase of copper levels in *M. restricta* cells, unlike what was observed in *S. cerevisiae* cells.

N,N,N′,N′-Tetrakis(2-pyridylmethyl)ethylenediamine (TPEN) is a high-affinity membrane permeable zinc chelator that has been used to investigate the effects of cellular zinc levels in multiple organisms, including fungi. Previous studies have suggested that ZPT dissociates into pyrithione and zinc salt once it is transported into the cytosol^21,22^. Therefore, we hypothesized that TPEN would chelate hyperaccumulated cellular zinc in the cytosol of *M. restricta* cells grown in the presence of ZPT, and that the inhibitory activity of ZPT against *M. restricta* would be reduced if TPEN and ZPT were added together. To test this hypothesis, we determined the minimal inhibitory concentration (MIC) of ZPT in the presence or absence of TPEN and found that the MIC of ZPT was reduced when it was added with TPEN (Table 1). These data confirmed our finding that ZPT increases cellular zinc levels in *M. restricta*. Additionally, we observed that ZPT caused swelling of *M. restricta* cells, and this abnormal morphological characteristic was restored by TPEN, but not by BCS. These data further support our findings (Supplementary Fig. S1). However, we should note that the addition of TPEN did not completely eliminate the antifungal activity of ZPT. These results imply that an increase in cellular zinc levels is not the only mechanism of antifungal action, but that pyrithione, which is dissociated from ZPT, might also inhibit *M. restricta*. Bathocuproine disulfonate (BCS) is a copper chelator, and its effect on the antifungal activity of ZPT against *M. restricta* was also tested. Unlike TPEN, the addition of BCS did not alter the MIC of ZPT, suggesting that the change in cellular copper levels was minimal.

**Table 1:** *In vitro* antifungal susceptibilities of *M. restricta* to ZPT with TPEN or BCS and pyrithione

	MIC (µM)
ZPT	2.5–5
ZPT + TPEN 25 µM	5–10
ZPT + TPEN 50 µM	10
ZPT + BCS 50 µM	2.5–5
ZPT + BCS 100 µM	2.5–5

.

### ZPT triggered a large change in the transcriptome and interfered with mitochondrial functions in *M. restricta*

To understand the mechanism of ZPT action in more detail, we investigated how ZPT affects the transcriptome of *M. restricta* cells, using the recently determined genome sequence of *M. restricta* KCTC 27527^26^. Transcriptomes of *M. restricta* cells grown in the presence of 5, 10, and 20 μM ZPT were analyzed by RNA sequencing (see Methods) and compared with those of cells grown in the absence of the compound. The minimum ZPT concentration of 5 μM was selected based on the MIC of the compound against *M. restricta*, and 2-fold increases in ZPT concentrations up to 20 μM were used to analyze the dose dependence of differentially expressed genes. The results of the transcriptome analysis showed that many genes were differentially expressed: 95, 490, and 724 genes were up or downregulated more than 2-fold in the cells grown in the presence of 5, 10, and 20 μM of ZPT, respectively, compared with the cells grown in medium without ZPT (Table 2 and Supplementary Table S2). Differential expression of few key genes were selected and validated by quantitative real time PCR (qRT-PCR; Supplementary Table S3).

**Table 2:** Number of genes regulated by ZPT treatment compared to no treatment.

ZPT treatment	The number of up-regulated genes					The number of down-regulated genes					Total
	2-Fold	3-Fold	4-Fold	>5-Fold	Total	2-Fold	3-Fold	4-Fold	>5-Fold	Total	
5 µM	14	5			19	64	10	1	1	76	95
10 µM	152	19	8	1	180	204	54	24	28	310	490
20 µM	221	50	10	13	294	267	80	29	54	430	724

**Figure 2:**
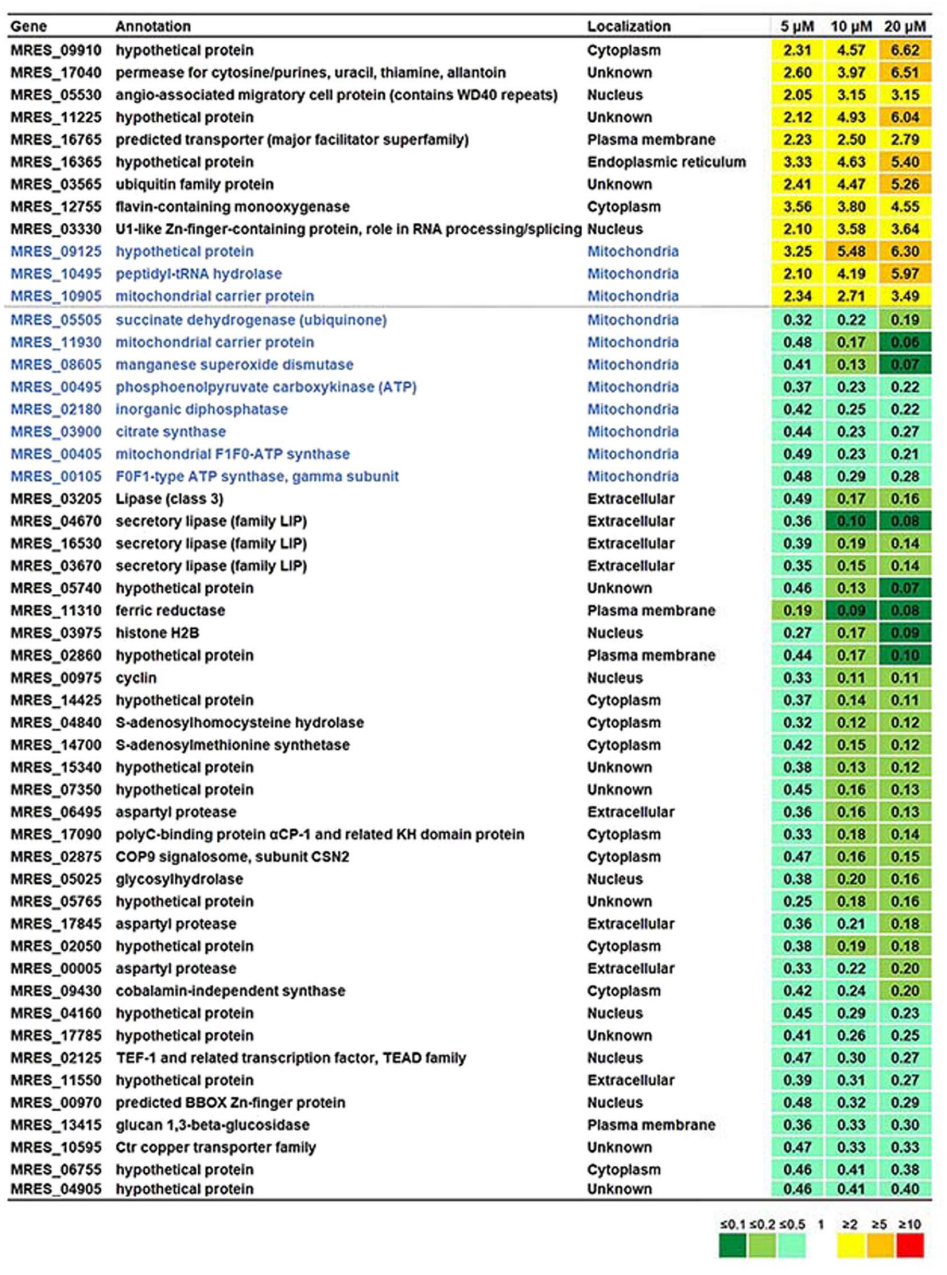
List of differentially expressed genes in ZPT-treated *M. restricta* cells. Genes that were more than 2-fold differentially up or downregulated in a dose-dependent manner in ZPT-treated *M. restricta* cells are shown. The values indicate the ratio of TMM of the corresponding gene in *M. restricta* cells grown in media containing different concentrations of ZPT vs. cells grown in medium without the compound.

We hypothesized that the genes that were directly affected by ZPT would show differential and dose-dependent expression in *M. restricta* cells grown in the media containing 5, 10, and 20 μM of ZPT. Applying highly stringent selection criteria, we found that, among the genes that were differentially expressed more than 2-fold, 12 and 40 genes were up and downregulated, respectively, in a dose-dependent manner in the cells grown in media containing different concentrations of ZPT. Moreover, we found that mitochondrial function might be one of the major targets of ZPT, because genes encoding mitochondrial proteins were the most enriched among the genes showing differential dose-dependent regulation (Fig. 2) such that 3 encoding mitochondrial proteins were upregulated while 8 were downregulated. In particular, the expression of MRES_05505 (succinate dehydrogenase), MRES_03900 (citrate synthase), MRES_00405 (ATP synthase subunit), and MRES_00105 (ATP synthase subunit), which are components of the tricarboxylic acid (TCA) cycle and electron transport chain, was downregulated significantly and dose-dependently.

**Figure 3:**
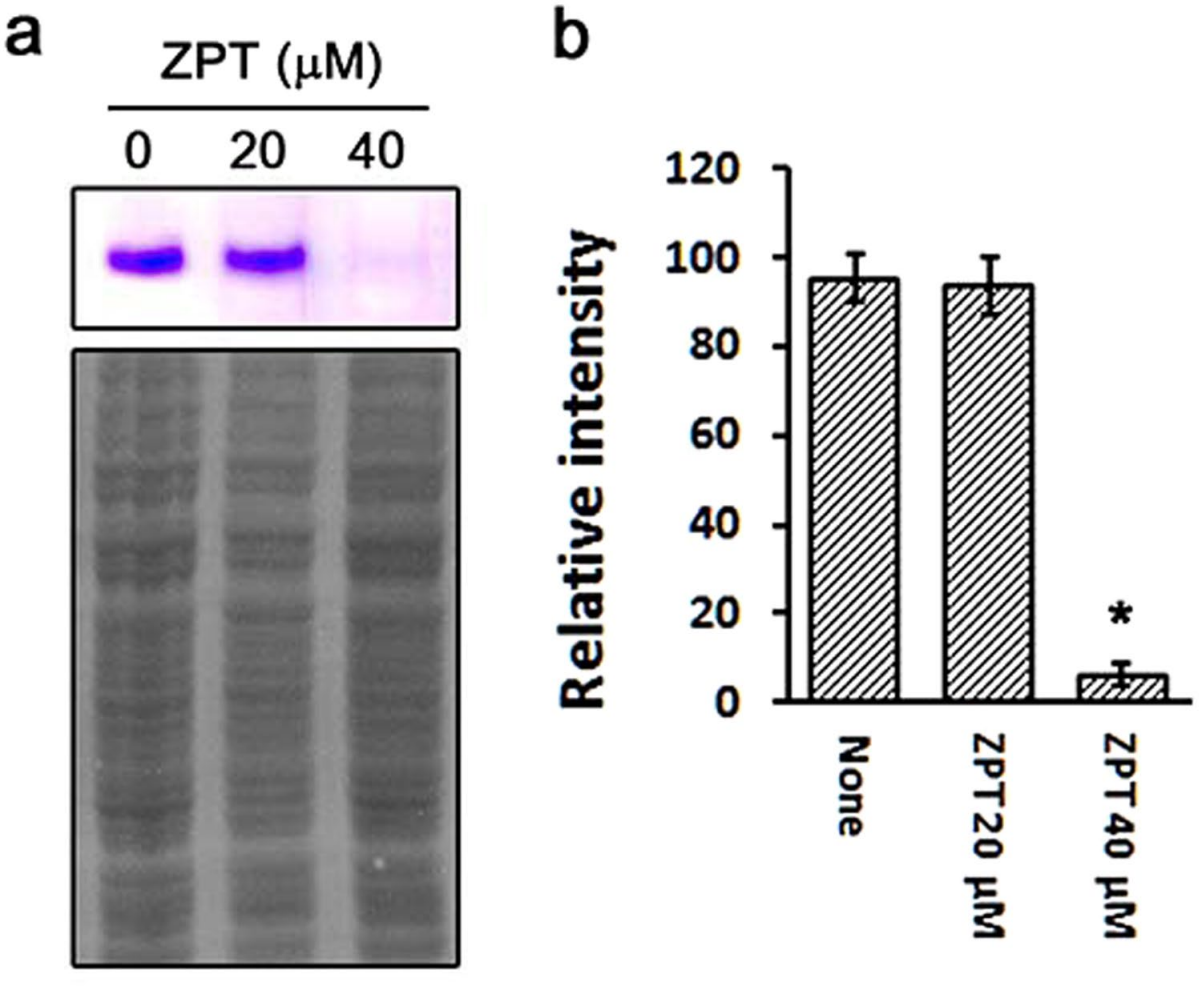
Activity of aconitase in ZPT-treated *M. restricta* cells. (**a**) Activity of aconitase in *M. restricta* KCTC 27527 cells grown in media containing 0, 20, or 40 µM of ZPT was assessed by gel-based aconitase assay (upper panel). As a loading control, the same samples were resolved on a polyacrylamide gel, transferred to nitrocellulose membranes, and stained with CPTA (lower panel). (**b**) The intensities of bands were quantified in ImageJ and compared. Values represent the average from three independent experiments with standard deviations (*p < 0.0001).

In a previous genome analysis, we identified two genes, MRES_16605 and MRES_08815 that are homologs of *ACO1* and *ACO2*, respectively, encoding aconitase in *S. cerevisiae*^26^. The current transcriptome analysis showed that *ACO1* homolog MRES_16605 was significantly downregulated in *M. restricta* cells grown in the presence of 5 and 10 μM ZPT, although the gene was not included in the list of dose-dependently regulated genes, because it was downregulated slightly less in the cells grown in medium containing 20 μM of ZPT. However, we confirmed reduced activity of aconitase in ZPT-treated *M. restricta* cells (Fig. 3). In agreement with this reduction in aconitase activity, the results of our transcriptome analysis showed that ZPT treatment upregulated a number of genes related to Fe-S cluster biosynthesis in *M. restricta* (Table 3). MRES_02625, MRES_04375, MRES_14620, and MRES_16875 were predicted to be required for Fe-S cluster assembly and were upregulated in a concentration-dependent manner in ZPT-treated *M. restricta* cells. We reasoned that upregulation of these genes occurred in the ZPT-treated cells to compensate for the inhibition of Fe-S cluster biosynthesis by ZPT. We should also note that these genes required for Fe-S biosynthesis were not included in the Fig. 2 of differentially expressed genes, because the changes in their expression in cells treated with 5 μM of ZPT were less than 2-fold.

**Table 3:** Expression of Fe-S cluster synthesis-related genes following ZPT treatment.

Gene name	Annotation	Fold (+ZPT/−ZPT)		
		5 µM	10 µM	20 µM
MRES_02625	Cytosolic Fe-S cluster assembly factor Nar1	1.81	3.03	3.47
MRES_04375	Fe-S cluster assembly protein Dre2	1.36	2.41	3.35
MRES_14620	Fe-S cluster assembly scaffold protein IscU	1.98	4.18	6.38
MRES_16875	Fe-S cluster scaffold homolog Nfu1	1.42	2.48	2.70

In addition to alterations in the expression of genes required for Fe-S cluster synthesis in mitochondria, MRES_08605, which encodes the homolog of *S. cerevisiae* Sod2, a manganese dependent mitochondrial superoxide dismutase, was found to be one of the most strongly dose-dependently downregulated genes in ZPT-treated *M. restricta* cells. It was shown that, in *S. cerevisiae*, *SOD2* transcription is significantly reduced with a decrease in heme synthesis, which occurs mainly within the mitochondria^28,29^. We, therefore, hypothesized that increased zinc accumulation in ZPT-treated *M. restricta* cells disrupted mitochondrial heme biogenesis and led to downregulation of *SOD2*. Taken together, our data suggested that ZPT interferes substantially with mitochondrial functions in *M. restricta* cells.

### ZPT altered the expression of genes involved in zinc and copper uptake in *M. restricta*

Altered cellular metal content in ZPT-treated *M. restricta* led us to investigate the differential expression of genes involved in zinc and copper transport. We first searched the genome of *M. restricta* for homologs of *S. cerevisiae* genes involved in transport of metals and found a few unique characteristics of metal transport systems in *M. restricta*. These include the presence of only a single copper transporter on the plasma membrane of *M. restricta*, whereas other fungi have at least two paralogous genes (*CTR1* and *CTR3* in *S. cerevisiae*^30,31^ and *CTR1* and *CTR4* in *Cryptococcus neoformans*, for example^32,33^). In addition, no vacuolar copper transporter, a homolog of *S. cerevisiae CTR2*^34^, was identified in the genome of *M. restricta*. In contrast, almost all homologs of zinc transporters, including two zinc transporters on the plasma membrane, were identified in the genome of *M. restricta* (Table 4). These findings suggested that *M. restricta* utilizes the conserved zinc transport system, with a limited number of genetic components used for copper transport in the fungus.

**Table 4:** Expression of Cu and Zn transporter genes following ZPT treatment.

	*S. cerevisiae*	*C. albicans*	*C. neoformans*	*M. restricta*	Annotation	Fold (+ZPT/−ZPT)		
						5 µM	10 µM	20 µM
Cu transporter	*CTR1*	*CTR1*	CNAG_07701 (*CTR1*)	No hit				
	*CTR2*	*CTR2*	CNAG_01872 (*CTR2*)	No hit				
	*CTR3*		CNAG_00979 (*CTR4*)	MRES_10595	Ctr copper transporter family	0.47	0.33	0.33
Zn transporter	*ZRT1*		CNAG_00895 (*ZIP1*)	MRES_03690/MRES_03695	ZIP zinc transporter, High-affinity zinc transport protein, Regulates intracellular zinc levels	0.60/1.11	0.64/0.87	0.65/0.65
	*ZRT2*	*ZRT1* *ZRT2*	CNAG_03398					
	*ZRT3*	*C2_02180W_A*	CNAG_02221	MRES_11910	ZIP zinc transporter, May act as a zinc-influx transporter	1.49	1.61	1.32
	*ZRC1*		CNAG_02806	MRES_04925	Region of a membrane-bound protein predicted to be embedded in the membrane, Zn2+ transporter Znt1 and related Cd2+/Zn2+ transporters (cation diffusion facilitator superfamily)	1.53	2.87	3.14
	*YKE4*		CNAG_02993	No hit				

We next analyzed the transcriptome data to determine expression patterns of genes involved in the transport of metals in ZPT-treated *M. restricta* cells. As shown in Table 4, the gene MRES_04925, which is a homolog of *S. cerevisiae ZRC1* and *COT1*, was upregulated in cells grown in the presence of 10 and 20 μM of ZPT. We should note that special attention was paid to MRES_04925 because of its possible function in zinc transport and homeostasis, although the gene was not included in the list of genes expressed differentially and dose-dependently. However, dose-dependent upregulation of the gene was observed in cells grown in the presence of 10 and 20 μM ZPT. Zrc1 and Cot1 are vacuolar zinc importers in *S. cerevisiae* that are responsible for zinc transport into vacuoles when intracellular zinc concentrations in a cell increase^35^. Increased expression of the *ZRC1* and *COT1* homolog in ZPT-treated *M. restricta* cells agreed with our findings of increased cellular zinc levels. We should also note that we observed a small reduction in the expression of MRES_03690 and MRES_03695, the homologs of *S. cerevisiae ZRT1* and *ZRT2*, which are plasma membrane transporters required for zinc uptake. The gene MRES_10595, the homolog of *S. cerevisiae CTR3*, was downregulated in ZPT-treated *M. restricta* cells. This data agreed with our findings in Fig. 1 showing slightly increased cellular copper levels in ZPT-treated *M. restricta* cells and implied that copper accumulation might also contribute inhibitory mechanism of ZPT at least partly.

### ZPT inhibits the expression of *M. restricta* lipase

**Figure 4:**
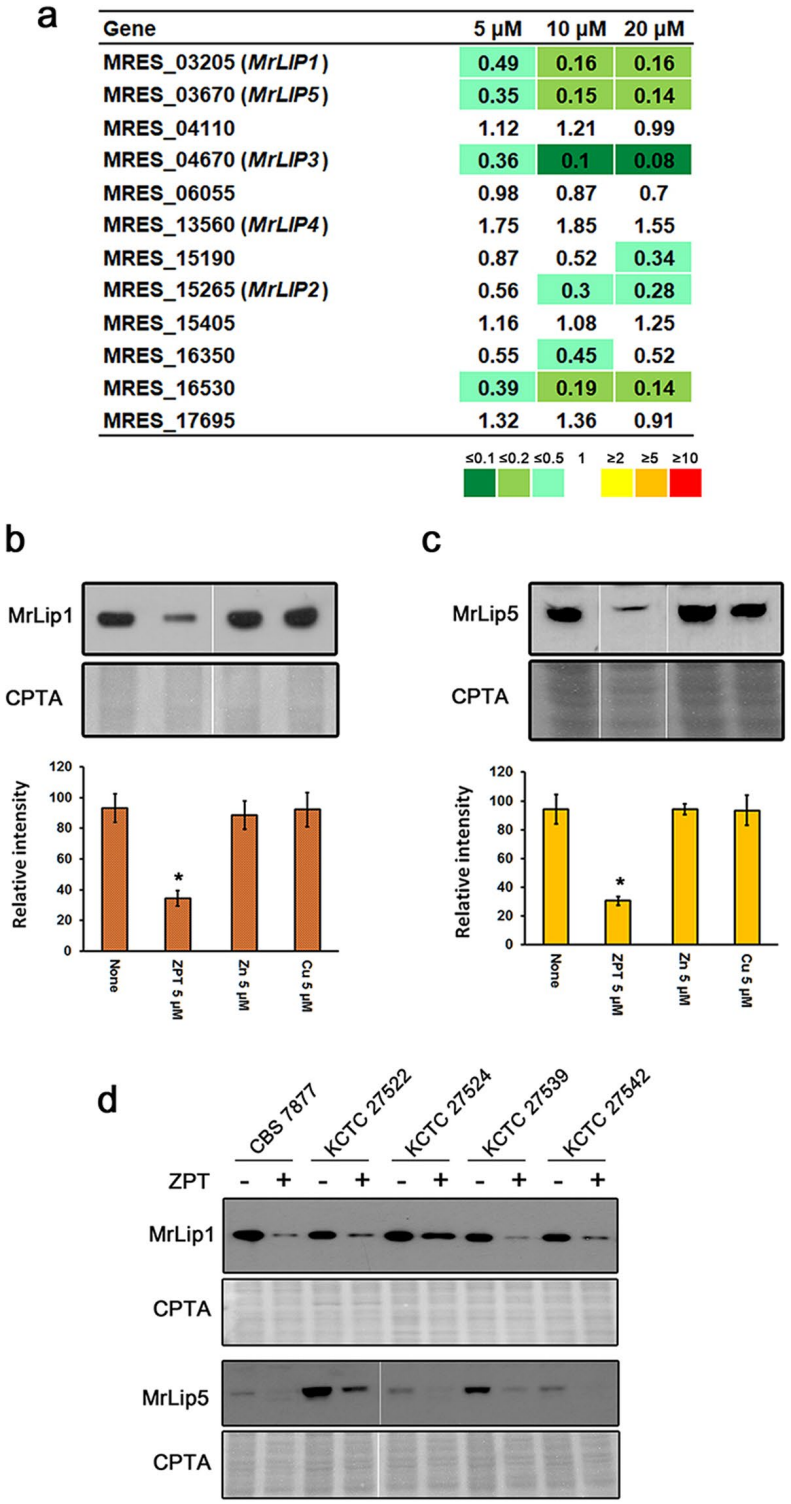
Inhibition of the expression of *M. restricta* lipases by ZPT. (**a**) Differential expression of 12 lipase genes in ZPT-treated *M. restricta* KCTC 27527 cells. The values indicate the ratio of TMM of the corresponding genes in *M. restricta* cells grown in media containing different concentrations of ZPT vs. cells in grown in medium without the compound. (**b**) Inhibition of MrLip1 expression in *M. restricta* cells grown in media containing 5 μM of ZPT, ZnCl_2_ or CuCl_2_ (upper panel). Relative intensities of expression were quantified in ImageJ and compared (lower panel). Values represent the average from three independent experiments with standard deviations (*p < 0.0001). The cropped blots are used in the figure, and the full-length blots are presented in Supplementary Fig. S2. (**c**) Inhibition of MrLip5 expression in *M. restricta* cells grown in media containing 5 μM of ZPT, ZnCl_2_ or CuCl_2_ (upper panel). Relative intensities of expression were quantified in ImageJ and compared (lower panel). Values represent the average from three independent experiments with standard deviations (*p < 0.0001). The cropped blots are used in the figure, and the full-length blots are presented in Supplementary Fig. S3. (**d**) Expression of MrLip1 and MrLip5 in various clinical isolates of *M. restricta* was assessed by western blot analysis. Nitrocellulose membranes were also stained with CPTA as a loading control. The cropped blots of are used in the figure, and the scanned full-length blots are presented in Supplementary Fig. S4. All western blot analysis were repeated at least three times, and representative images were presented.

Another group of genes that showed a significant differential response to ZPT was lipases. We previously identified 12 lipase genes in the genome of *M. restricta* KCTC 27527^26^. Among them, MRES_03205 (*MrLIP1*), MRES_03670 (*MrLIP5*), MRES_04670 (*MrLIP3*), and MRES_16530 were downregulated more than 2-fold in a dose-dependent manner in response to ZPT treatment. Putative lipase-encoding genes, MRES_15190 and MRES_15265 (*MrLIP2*), were also downregulated in a dose-dependent manner, but slightly less than 2-fold with 5 and 10 μM ZPT. In total, the expression of 6 out of 12 lipases was downregulated in ZPT-treated cells (Fig. 4a). We paid special attention to the downregulation of lipase genes, because these enzymes are believed to play key roles in the pathogenesis of *Malassezia* species^2,26,27,36^. Among them, *MrLIP5* was of particular interest, because it was identified as a major lipase-encoding gene and the most frequently expressed gene in the scalps of patients with dandruff^26^. To confirm downregulation of lipases by ZPT, we performed a western blot analysis using antisera specific to MrLip1 and MrLip5 and found that the decreased transcript levels observed in our transcriptome analysis reflected significantly reduced protein levels (Fig. 4b,c). The results of the western blot analysis also suggested that reduced expression of lipases was not a response to an increase in zinc or copper in the medium. Moreover, downregulation of the expression of lipases, MrLip1 and MrLip5 in particular, by ZPT has been observed in other clinical isolates of *M. restricta*, eliminating the possibility of a strain-specific inhibitory effect of ZPT on lipase expression (Fig. 4d). These results indicated that ZPT had an inhibitory effect on lipase expression across various clinical strains of *M. restricta*, suggesting that this is a general mechanism by which the compound limits *M. restricta*.

## Discussion

In the present study, we aimed to understand the mechanism of action of ZPT against *M. restricta* and found that ZPT treatment significantly increased cellular zinc levels in the fungus along with a small increase of cellular copper levels. A reduction in the antifungal activity of ZPT on *M. restricta* by TPEN, an cellular zinc chelator, but not by BCS, a copper chelator, supports our observation that ZPT treatment mainly causes increase in cellular zinc levels. These results were different from what was found in a previous study with *S. cerevisiae*, which showed that the addition of BCS rescued the growth of fungal cells treated with ZPT^25^ and suggested that the mechanism of action of ZPT against *M. restricta* is different from that against *S. cerevisiae*. In *S. cerevisiae*, two plasma membrane proteins, Ctr1 and Ctr3, are responsible for high-affinity copper uptake, and Ctr2 is involved in copper export through the vacuolar membrane. The previous study showed that ZPT triggered a 0.08~0.23-fold decrease in *CTR1*, supporting the idea that cellular copper levels are increased in response to the compound in *S. cerevisiae*. However, we discovered that *M. restricta* lacks homologs of *S. cerevisiae* Ctr1 and Ctr2. Instead, *M. restricta* may utilize a single copper transporter, a homolog of *S. cerevisiae* Ctr3, to transport copper through the plasma membrane, implying that the copper transport system of this fungus is different from that of *S. cerevisiae*. Nevertheless, the downregulation of the *CTR3* homolog in ZPT-treated *M. restricta* suggested that cellular copper levels were indeed increased slightly in the cells and implied that the small increase of copper accumulation might also contribute to the growth inhibition by ZPT. Unlike the copper transport system, *M. restricta* possesses highly-conserved fungal zinc transport systems. We noticed significantly upregulated expression of the homolog of *S. cerevisiae* vacuolar zinc transporters Zrc1 and Cot1 in ZPT-treated *M. restricta* cells, suggesting an increase in cellular zinc levels in the fungus.

Our transcriptome analysis revealed that ZPT inhibits Fe-S cluster synthesis in *M. restricta*, a result similar to that obtained for ZPT-treated *S. cerevisiae* cells^24,25^. Fe-S cluster synthesis is one of the main iron metabolic processes that occurs in mitochondria, and increases in cellular zinc levels in response to ZPT treatment might be the main cause of Fe-S cluster synthesis inhibition in *M. restricta* We should note that, although the change was minimal, a small increase of cellular copper levels might also contribute to inhibits Fe-S cluster synthesis in the ZPT-treated *M. restricta* cells. Several previous studies have suggested that hyper-accumulation of cellular zinc causes mitochondrial dysfunction that is associated with the accumulation of reactive oxygen species in the organelle, which leads to a reduction in ATP production, because this accumulation interferes with glycolysis, tricarboxylic acid cycle, and electron transport chain^37–39^. Our data showed differential expression of a number of genes involved in mitochondrial functions, including the activities of the TCA cycle and electron transport chain, as well as significantly reduced aconitase activity in ZPT-treated *M. restricta*. In general, fungal strains that are deficient in Fe-S cluster synthesis increase expression of the genes responsible for iron uptake in the plasma membrane to compensate for the iron metabolism deficiency^40–42^. ZPT-treated *S. cerevisiae* cells also showed increased expression of *FET3*, a component of the high-affinity reductive iron uptake system in the plasma membrane. Interestingly, we found that components of this system such as an iron permease and a ferroxidase, which have been identified in other fungi such as *S. cerevisiae*, *Candida albicans*, *Cryptococcus neoformans*, and *Aspergillus fumigatus*, are largely missing in the genome of *M. restricta*. However, we found that *M. restricta* possesses the genes required for siderophore synthesis and observed that MRES_12755, a homolog of *A. fumigatus SIDA*, which encodes l-ornithine *N*^5^-oxygenase in the siderophore synthesis pathway^43^, was upregulated in dose-dependent manner, supporting our hypothesis that Fe-S cluster synthesis was significantly distorted in *M. restricta* in response to ZPT.

Our study revealed that expression of *SOD2*, the gene encoding a manganese dependent mitochondrial superoxide dismutase, may be directly or indirectly inhibited by ZPT in *M. restricta*. The *SOD2* gene was one of the most strongly downregulated genes in ZPT-treated *M. restricta*, an observation that led us to speculate that the significant downregulation of Sod2 may contribute to decreased Fe-S cluster synthesis and reduced aconitase activity in the fungus. We developed this hypothesis because a number of studies showed that mitochondrial reactive oxygen species were increased in a mutant lacking *SOD2*, and that *SOD2*-mutant became hypersensitive to oxidative stress^44–46^. How ZPT triggers downregulation of *SOD2* is not yet clear. However, a connection between cellular zinc toxicity and heme synthesis in other fungi may explain this phenomenon. Zinc toxicity causes reduced heme production in *A. fumigatus*^47^, and deficiency in heme synthesis significantly reduced *SOD2* transcript levels in *S. cerevisiae*^28^. Therefore, we speculated that ZPT increased cellular zinc toxicity, which in turn reduced heme synthesis, followed by a reduction in *SOD2* transcription in ZPT-treated *M. restricta* cells. Although an additional study is required to test this hypothesis, we suppose that the increase in cellular zinc introduced by ZPT triggered significant downregulation of *SOD2*, which disrupted Fe-S cluster synthesis and led mitochondrial dysfunction in *M. restricta*.

A recent genome analysis revealed that almost all *Malassezia* species, including *M. restricta*, lack the fatty acid synthase gene in their genomes. Instead, they possess numerous genes encoding lipases that acquire fatty acids as a nutrient source from sebum on host skin^48–50^. After hydrolysis of exogenous lipids in sebum by lipases secreted by *Malassezia*, free fatty acids (FFA) may be released and transported into the fungal cells, suggesting that lipases play essential roles in the physiology of the fungi. The increased levels of FFA because of these lipases on skin surfaces lead to abnormal functioning of epidermal keratinocytes, which causes skin irritation^13,51,52^. Moreover, oleic acid, an FFA generated by extracellular lipases of *Malassezia*, has been shown to induce scalp flaking and skin irritation in susceptible subjects^2^. Previously, we identified 12 lipase-encoding genes in the genome of *M*. *restricta* and found that, among these genes, MRES_03670 (*MrLIP5*) is expressed most frequently on the surfaces of scalps of patients with dandruff, suggesting the importance of this lipase in fungus-host interactions. The present study revealed that ZPT treatment downregulated four out of 12 lipase genes (MRES_03205 [*MrLIP1*] MRES_03670 [*MrLIP5*], MRES_04670 [*MrLIP3*], and MRES_16530) in a dose-dependent manner, suggesting that inhibition of lipase expression is one of the main mechanisms by which ZPT inhibits *M. restricta*. Downregulation of lipase genes observed in our transcriptome analysis was confirmed by western blot analysis, which showed a reduction in the levels of the corresponding lipases. Reduced expression of lipases in response to ZPT treatment was also observed in several other clinical isolates of *M. restricta*, indicating that inhibition of lipase expression is a common mechanism of ZPT action against *M. restricta*. We should note that downregulation of *MrLIP5* by ZPT is particularly important because of the possible involvement of the corresponding protein in skin surface pathogenesis^26^. However, the upstream regulator that inhibits the expression of lipases in response to ZPT treatment still needs to be identified. Taken together, our results suggest that at least three inhibitory mechanisms are associated with the action of ZPT against *M. restricta*: (i) an increase in cellular zinc levels, (ii) inhibition of mitochondrial functions, and (iii) a decrease in lipase expression.

## Methods

### Strains and culture conditions

*M. restricta* KCTC 27522, KCTC 27524, KCTC 27527, KCTC 27539, and KCTC 27542 were clinically isolated in our previous study and used here, along with the type strain *M. restricta* CBS 7877^53,54^. *Malassezia* strains were grown in Leeming and Notman agar (LNA) solid medium (0.5% glucose, 1% peptone, 0.01% yeast extract, 0.8% bile salt, 0.1% glycerol, 0.05% glycerol monostearate, 0.05% Tween 60, 1.2% agar, 0.5% whole fat cow milk, and 170 µg/mL chloramphenicol) at 34 °C for 3 days^55^. *S. cerevisiae* W303 was grown in YPD medium (1% yeast extract, 2% peptone, and 2% glucose)^56^.

### Determination of the ZPT susceptibility of *M. restricta*

We used a previously reported method with modifications to determine the susceptibility of *M. restricta* KCTC 27527 to ZPT (Sigma, St. Louis, MO, USA)^57^. Briefly, a stock solution of ZPT at a final concentration of 20 µM was prepared and used for the susceptibility assay. The stock solution was serially diluted 2-fold with LNA medium and introduced into the wells of a 24-well plate*. M. restricta* cells, pre-grown in LNA medium, were inoculated using a cotton swab into each well of the 24-well plate containing different concentration of ZPT and incubated at 34 °C for 3 days. Minimal inhibitory concentrations (MICs) were determined, based on the lowest concentration at which there was no visible growth of the cells compared to that in medium without ZPT. The susceptibility assay was independently performed at least four times.

### Determination of intracellular metal content

Total intracellular zinc, copper, iron, and manganese levels were determined by inductively coupled plasma-atomic emission spectroscopy (ICP-AES). *M. restricta* KCTC 27527 cells were cultured for 3 days and transferred to fresh LNA medium containing ZPT (0, 5, 10, or 20 µM) at 34 °C for 6 h. Cells were harvested, washed with washing buffer (50 mM Tris-HCl [pH 6.5] and 10 mM ethylenediaminetetraacetic acid three times and lyophilized, as described in a previous report^25^. The cell mass was digested with 5 mL of HNO_3_ and 3 mL of H_2_O_2_ using a microwave digestion system, START D (Milestone, Sorisole, Italy), and an ICP-AES analysis was performed using an Optima 5300 DV system (PerkinElmer, Waltham, MA, USA). To measure the concentrations of metals in *S. cerevisiae* W303, we followed methods described by Reeder *et al*.^25^.

### RNA isolation and transcriptome analysis

*M. restricta* KCTC 27527 cells were cultured in LNA medium at 34 °C for 3 days and transferred to fresh LNA medium containing ZPT (0, 5, 10, or 20 µM). The cells were incubated at 34 °C for 6 h and harvested for RNA extraction. Total RNA was extracted using an RNeasy Mini Kit (Qiagen, Hilden, Germany) following the manufacturer’s instructions. The quality of RNA was evaluated using an Agilent 2100 Bioanalyzer (Agilent Technologies, Santa Clara, CA, US). Libraries for RNA sequencing were prepared with a TruSeq Stranded mRNA Sample Prep Kit (Illumina, San Diego, CA, US), according to the manufacturer’s protocol, and RNA sequencing was performed on an Illumina HiSeq platform to generate 100-bp paired-end reads. Quality-filtered reads were aligned to the reference-genome sequence using bowtie2 v2.2.1^58^. Mapped reads were counted by feature Counts in Subread package v1.4.3^59^, and the relative transcript abundance was trimmed mean of M-values (TMM)-normalized by edgeR^60^. Differential expression of few key genes were selected and validated by qRT-PCR using primers listed in Supplementary Table S4 and a 7500 system (Applied Biosystems, Foster, CA, USA). The actin gene (MRES_06100) was used as a reference.

### Aconitase assay

Aconitase activity was measured, as described previously^61^. Briefly, *M. restricta* KCTC 27527 cells were cultured in LNA medium at 34 °C for 3 days, transferred to fresh LNA medium, incubated with or without ZPT (20 and 40 µM) at 34 °C for 16 h, and then harvested. The cells were lysed with Triton-citrate lysis buffer containing 22.9 mM Tris-HCl pH 7.5, 36.6 mM KCl, 1% Triton X-100, 2 mM sodium citrate, 0.6 mM MnCl_2_, 1 mM dithiothreitol, 1 mM phenylmethylsulfonyl fluoride, and a 1% protease inhibitor cocktail. The polyacrylamide gel used to assess aconitase activity comprised a separating gel containing 8% acrylamide, 132 mM Tris-base, 132 mM borate and 3.6 mM citrate, and a stacking gel containing 4% acrylamide, 67 mM Tris-base, 67 mM borate, and 3.6 mM citrate. The running buffer contained 192 mM glycine, 3.6 mM citrate, and 25 mM Tris-HCl (pH 8.3). A total of 20 µg of protein extract from each sample was separated by electrophoresis at 160 V and 4 °C for 2.5 h. The aconitase activity was visualized by staining the gel with 100 mM Tris-HCl (pH 8.0), 1 mM nicotinamide adenine dinucleotide phosphate, 5 mM MgCl_2_, 2.5 mM *cis*-aconitic acid, 1.2 mM methylthiazolyldiphenyl-tetrazolium bromide, 0.3 mM phenazine methosulfate, and 5 U/mL isocitrate dehydrogenase in the dark at 37 °C for 20 min. To show equal loading of each sample, the same amount of protein extract on the nitrocellulose membrane was stained with copper phthalocyanine-3,4ʹ,4ʹʹ,4ʹʹʹ-tetrasulphonic acid tetrasodium (CPTA).

### Protein extraction and western blot analysis

*M. restricta* strains were cultured in LNA medium at 34 °C for 3 days and transferred to fresh LNA medium with or without 5 µM of ZPT, Zn, or Cu. The cells were incubated at 34 °C for an additional 16 h, harvested, and suspended in cell lysis buffer containing 140 mM NaCl, 50 mM 4-(2-hydroxyethyl)-1-piperazineethanesulfonic acid-KOH (pH 7.5), 1 mM ethylenediaminetetraacetic acid, 1% Triton X-100, 0.1% Na-deoxycholate, and 1 mM phenylmethylsulfonyl fluoride^62^. Total proteins were extracted by adding 600 µL of glass beads (0.5 mm) and vortexing the solution for 3 min. The western blots were performed using anti-MrLip1 and anti-MrLip5 sera, produced in our previous study^53^, as primary antibodies and goat anti-rabbit IgG-horseradish peroxidase (Santa-Cruz Biotechnology, Dallas, TX, USA) as the secondary antibody.

### Statistical analysis

Statistical analysis for differences between each sample were calculated using the GraphPad QuickCalcs Web site (https://www.graphpad.com/quickcalcs/ttest1.cfm) with unpaired t-tests. p < 0.05 was accepted to mean a statistically significant difference.

## Electronic supplementary material

Supplementary Information

## Data Availability

The datasets generated and analyzed during the current study are available in the Gene Express Omnibus (GEO) repository, under the accession number GSE112036.
