# Supplementary material for: Understanding the Mechanism of Action of the Anti-Dandruff Agent Zinc Pyrithione against Malassezia restricta
Source: Sci Rep. 2018 Aug 14;8:12086. doi: 10.1038/s41598-018-30588-2 (PMC6092343; doi:10.1038/s41598-018-30588-2)
Supplement: Supplementary file 1 — Supplementary Information [file 41598_2018_30588_MOESM1_ESM.pdf]

Understanding the Mechanism of Action of the Anti-Dandruff Agent Zinc Pyrithione against *Malassezia restricta*

Minji Park[1], Yong-Joon Cho[2], Yang Won Lee[3, 4,*], Won Hee Jung[1,*]

[1]Department of Systems Biotechnology, Chung-Ang University, Anseong, 17546, Korea

[2]Korea Polar Research Institute, Incheon, 21990, Korea

[3]Department of Dermatology, School of Medicine, Konkuk University, Seoul, 05029, Korea

[4]Research Institute of Medicine, Konkuk University, Seoul, 05029, Korea

Short title: Mechanism of Action of Zinc Pyrithione

Keywords:

Anti-dandruff, *Malassezia*, mechanism of action, zinc pyrithione

[*] **Correspondence to:**

Won Hee Jung, PhD, Department of Systems Biotechnology, Chung-Ang University, Anseong, 17546, Korea, e-mail: whjung@cau.ac.kr

Yang Won Lee, MD, PhD, Department of Dermatology, School of Medicine, Konkuk University, Seoul, 05029, Korea, e-mail: 20050078@kuh.ac.kr

Supplementary Table S1. Cellular metal analysis in ZPT treated *S. cerevisiae* W303

	Fe	Zn	Mn	Cu
- ZPT	199.50 ± 2.31	413.55 ± 9.01	6.74 ± 0.12	2.48 ± 0.16
+ ZPT 1.5 μM	172.20 ± 1.34	392.55 ± 3.85	6.52 ± 0.06	6.34 ± 0.10
+ ZPT 3 μM	183.29 ± 3.13	314.35 ± 4.34	6.93 + 0.11	9.38 ± 0.06

*All values represent weight (μg) per dry weight (g).

Supplementary Table S3. **Validation of RNA Sequencing data by qRT-PCR**

Gene	Description	Fold (ZPT 20 μM/ZPT 0 μM)	
		RNA Sequencing	qRT PCR
MRES_03205	Lipase (MrLip1)	0.16	0.49±0.07
MRES_03670	Lipase (MrLip5)	0.14	0.32±0.08
MRES_03690	Zinc-regulated transporter 1	0.65	0.48±0.06
MRES_04670	Lipase (MrLip3)	0.08	0.37±0.07
MRES_04925	Zn2+ transporter Znt1 and related Cd2+/Zn2+ transporters	3.14	4.30±0.15
MRES_08605	Iron/manganese superoxide dismutases	0.07	0.16±0.03
MRES_11310	Ferric reductase	0.19	0.19±0.01
MRES_14620	Fe-S cluster assembly scaffold protein IscU	6.38	11.13±0.95
MRES_16530	Lipase	0.14	0.27±0.02

Supplementary Table S4. Primers used for qRT-PCR

Primer	Target gene	Sequence (5'-3')
qRT.MRES_06100.F	MRES_06100	GCACCACACGTTCTACAACGA
qRT.MRES_06100.R		GTGAGAAGGACAGGGTGCTCTT
qRT.MRES_03205.F	MRES_03205	GCTGCGCGGCTCTTGTA
qRT.MRES_03205.R		TCGAACTACCGGCATGAAAGT
qRT.MRES_03670.F	MRES_03670	GCCTCAAGGCTGGTCAGAAC
qRT.MRES_032670.R		TTGAGCACGTGAAGCTTGGT
qRT.MRES_03690.F	MRES_03690	ATGAGCTTGGGCAGGAGTGT
qRT.MRES_03690.R		CGTACGCCATCGGGTAATTC
qRT.MRES_04670.F	MRES_04670	CACGGCGCCGACATTC
qRT.MRES_04670.R		ACGCGTGGCCCATCAC
qRT.MRES_04925.F	MRES_04925	CGCTGTGCGCCTCGTAA
qRT.MRES_04925.R		GCTGCCATCCATACGAATACTG
qRT.MRES_08605.F	MRES_08605	AGCCAGGGCGGTGGTAA
qRT.MRES_08605.R		CCTTTTCGATGGCCTGCTT
qRT.MRES_11310.F	MRES_11310	GCTGGCGTCCAGGACAAC
qRT.MRES_11310.R		TGCGTAGCAGCCATTCCA
qRT.MRES_14620.F	MRES_14620	CAACGTCGGCTCGTTTGC
qRT.MRES_14620.R		GCACCAACGAGGCCGATA
qRT.MRES_16530.F	MRES_16530	TTCACTGCGCTGGGAACA
qRT.MRES_16530.R		GGGTTGTAGAACCTGTCGTCATC

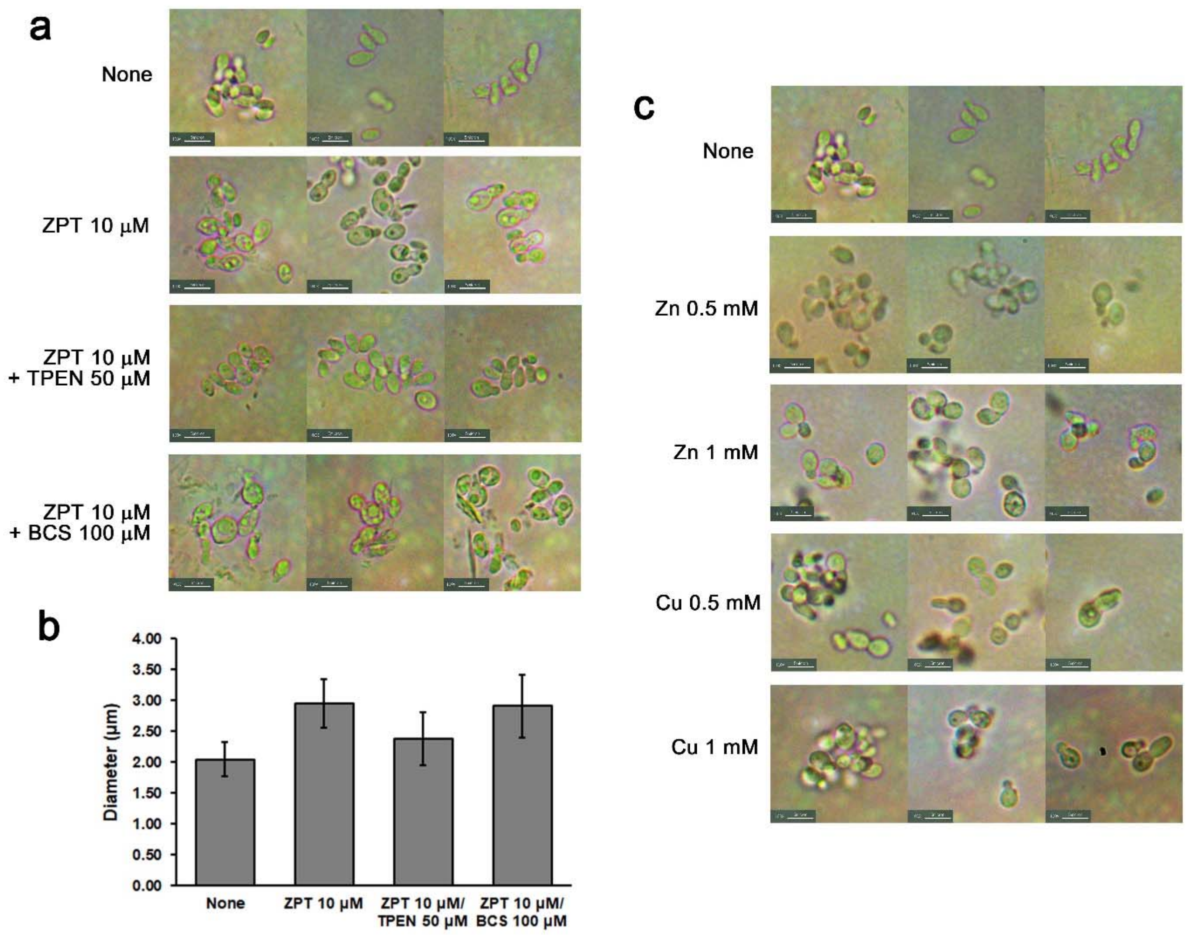

Supplementary Figure S1. (a) Morphology of *M. restricta* KCTC 27527 cells in the absence or presence of 10 µM ZPT with 50 µM TPEN or 100 µM BCS. (b) The diameter of 100 cells was measured, and averages were compared. (c) Morphology of *M. restricta* KCTC 27527 cells in the presence of 0.5 mM and 1 mM $ZnCl_2$ and $CuCl_2$. The scale bar represents 5 µm.

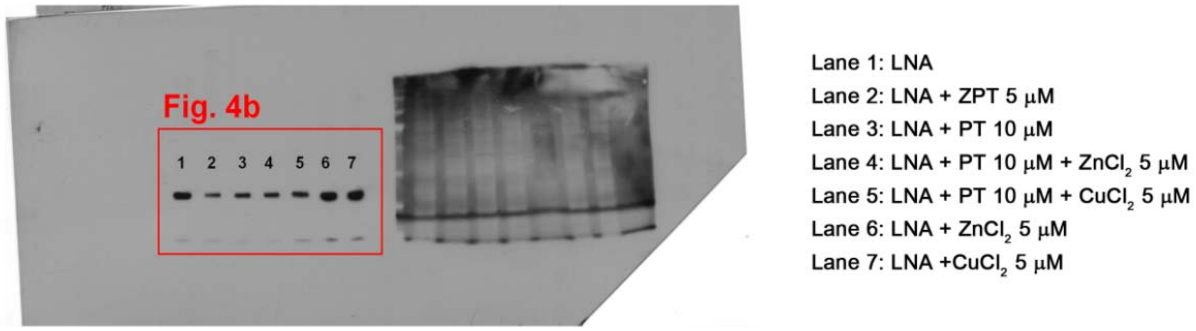

Supplementary Figure S2. The scanned full-length blots of the MrLip1 expression in *M. restricta* KCTC 27527. In the original blots, we selected lane 1, 2, 6 and 7 to show ZPT effect on MrLip1 and cropped the blots.

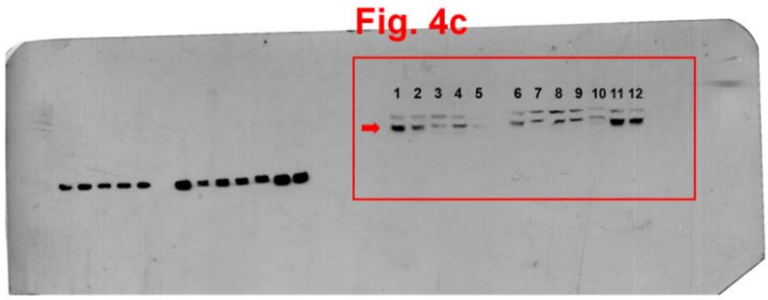

Lane 1: LNA
Lane 2: LNA + ZPT 2.5 μM
Lane 3: LNA + ZPT 5 μM
Lane 4: LNA + ZPT 10 μM
Lane 5: LNA + ZPT 20 μM
Lane 6: LNA
Lane 7: LNA + ZPT 5 μM
Lane 8: LNA + PT 10 μM
Lane 9: LNA + PT 10 μM + $ZnCl_2$ 5 μM
Lane 10: LNA + PT 10 μM + $CuCl_2$ 5 μM
Lane 11: LNA + $ZnCl_2$ 5 μM
Lane 12: LNA +$CuCl_2$ 5 μM

Supplementary Figure S3. The scanned full-length blots of the MrLip5 expression in *M. restricta* KCTC 27527. In the original blots, we selected lane 1, 7, 11 and 12 to show ZPT effect on MrLip5 and cropped the blots.

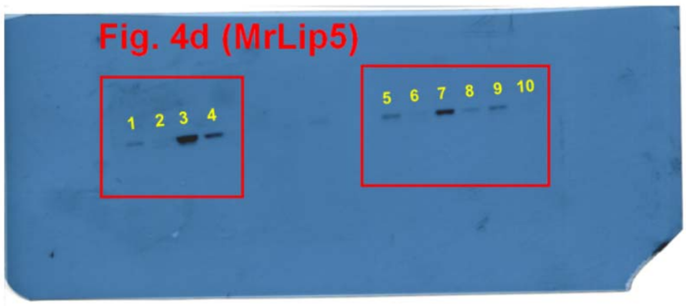

Lane 1: CBS 7877, -ZPT
Lane 2: CBS 7877, +ZPT
Lane 3: KCTC 27522, -ZPT
Lane 4: KCTC 27522, +ZPT
Lane 5: KCTC 27524, -ZPT
Lane 6: KCTC 27524, +ZPT
Lane 7: KCTC 27539, -ZPT
Lane 8: KCTC 27539, +ZPT
Lane 9: KCTC 27542, -ZPT
Lane 10: KCTC 27542, +ZPT

Supplementary Figure S4. The scanned full-length blots of the MrLip5 expression in in various clinical isolates of *M. restricta*. We cropped and assembled the blots.
